# On the Diseases and Injuries of Arteries, with the Operations Required for Their Cure

**Published:** 1830-10-01

**Authors:** 


### On the Diseases and Injuries of Arteries, with the Operations required for their Cure. By *G. J. Guthrie*, F.R.S. &c. &c. &c. Octavo, pp. 408, London, 1830.

[Second and concluding Article.]

In the last quarterly number of this Journal, we gave a pretty full analysis of the portion of Mr. Guthrie's work which comprises the injuries to which the arteries are exposed, and the most appropriate methods of treating them. It now remains for us to notice the first and more copious part, which is dedicated to the important subject of aneurism. It might be thought that the labours of the many eminent individuals, whose researches have thrown a halo of glory on the surgical annals of the close of the last, and commencement of the present century, would have rendered unnecessary any further investigations on the diseases of the arteries. Guattani, Lancisi, and Scarpa in Italy; Pelletan, Corvisart, Dessault and Laennec in France; and more than all the galaxy of genius which our native land can boast, Hunter, Abernethy, Allan Burns, Home, Cooper, the Bells, Hodgson, and Guthrie, have left, one would imagine, little more to desire on the subject that exercised their observation and their talents.

But such is the imperfection of the human mind, or the inherent difficulties of the sciences which it creates and which still perplex and baffle their creator, that the accidental or morbid lesions of the nervous system are yet in need of further elucidation. What physiology, anatomy, and the keenest observation could do, may be fairly said to have been done already, but our forefathers and predecessors were in want of a weapon that peculiarly distinguishes us, we mean the extensive employment of post-mortem investigations. It is like the invention of gunpowder in fiercer warfare than our own; and will simplify tactics, if it does not save lives. We have for some time been convinced that the connexion beween the diseases of the heart and the arteries is one that requires the most careful, close, and extended study. It must be the work of some years spent at the bed-side, in the hospital, and in the dead-house. If a man of moderate talents will devote his time to this investigation, and throw on it the lights which auscultation certainly affords, we prophecy, and we do it with a little knowledge of the subject, that the result will be highly beneficial to his profession, his species, and himself. Such a plan as we have chalked out must require some years to be properly put into effect, but much may, and we doubt not will, be done by able and observant surgeons in the interim.

If some of our readers imagine that we already know as much as is likely to be practically useful, we beg in reply to direct their attention to the various papers on the heart and circulation, which appear from time to time in the periodical publications. So little of certainty is there on these topics, that Harvey and Laennec are at variance on the rythm of the heart, and later writers, like Dr. Williams and Dr. Corrigan, hold opinions differing from either. Can it be said that a state of doubt and difficulty such as this, is a safe foundation whereon to rear pathological and diagnostic structures? Or can it be denied that very much is yet wanted to arrange our ideas and to settle our theories? We do not expect a millenium—far from it; this will only be found in the brains of enthusiasts, or the pages of radical reformers. But we do expect, or desire at all events, an epoch in which our principles, with regard to the heart and arteries, shall become more stable and settled, and our facts established on less disputable evidence. But waiving any further remarks of this kind, we shall proceed to the consideration of the volume before us. We have given a tolerably full analysis of the portion which treats of the accidental injuries of arteries, because we fear the worse informed members of the profession have but very erroneous notions on the subject, and inflict no trifling quantum of mischief on their patients. It is somewhat different with respect to aneurism; not only are the common ideas of its nature more correct, but its management and treatment generally fall to the province of experienced men. This is not always the case, we allow, for we daily read of instances of aneurism, in which lancets have been thrust into the tumour by ignorant practitioners, under the impression that its contents were purulent. It cannot be supposed that any surgeon of the present day could write a treatise upon this disease, containing *much* more than is found in the works of Scarpa and Hodgson. Too short a time has elapsed since the appearance of those admirable volumes, to permit the accumulation of any great store of addenda. On this account we must be excused from entering on a full or complete analysis of Mr. Guthrie's observations; our object will be rather to communicate to our readers the particular remarks on received opinions which his judgment may direct; the important facts which his experience may have amassed; or the novel views which judgment, experience, and reasoning combined may elicit.

After giving a very good anatomical description of the structure and coats of an artery, our author makes the following very judicious remarks:—

"This capability for contraction, on the application of certain stimuli, gave rise to the opinion of arteries being muscular, but which has been disputed on account of their not being found by chemical analysis to possess the same component parts, and particularly the same quantity of fibrin, as muscles. The terms contractility, retractility, tonicity, &c. have therefore been preferred to that of muscularity. It is however a mere difference about a name; no one in the present day doubting or disputing the fact of the capability of an artery to alter its dimensions through the agency of a power distinct from elasticity, and which may with propriety be termed contractility. It would indeed be taking a very limited view of nature, to suppose that every thing possessing contractile powers must be muscular, because we know that a muscle contracts." 5.

This appears to us to be the true state of the case, and Mr. Guthrie's manner of advocating it is so clear as not to require further comment or elucidation. Passing over some physiological points, we arrive at the alterations of structure to which arteries are liable, a topic of the greatest importance in every point of view. Aneurism, unless it be the consequence of accidental violence, seldom takes place in an artery perfectly sound. Before the vessel yields to the impulse of the blood against its sides, in other words, before it undergoes dilatation, its elasticity and contractility are usually materially impaired by the deposition of atheromatous matter between its internal and middle coats, or conversion of one or both into membranes of a nearly similar character. It thus becomes a matter of some moment to understand, if possible, the laws which govern the formation of those depositions. From the earliest period of the cultivation of morbid anatomy, dissectors have ever longed to establish the existence of arteritis, nay more, to believe that it is a disease of frequent occurrence and peculiar characters. Hitherto their speculations have proved abortive; and not only are the symptoms of this disease unknown, but men of experience are dubious of its existence, or at all events have never witnessed its occurrence. For our own parts we place no confidence whatever in the descriptions of authors on the subject, and out of the number of bodies we have seen examined, we must say that we have never been sufficiently fortunate to discover arteritis. The *staining* of the inner tunics of the blood vessels, and more particularly of the aorta, has been often mistaken for inflammation ; but after the masterly exposition of the error by Laennec, and the various papers that have appeared from time to time on the Continent and in this country, papers to which we have attracted the notice of our readers, no well-informed experienced man would in future commit such a blunder. Mr. Guthrie divides inflammation of arteries into phlegmonous and erysipelatous, denominations which, though certainly objectionable in an abstract sense, are yet convenient when taken in his own. By the phlegmonous is understood that healthy action which seals the cut or otherwise injured artery with coagulable lymph, obstructing the channel of the vessel it is true, but effectually preventing the occurrence of hæmorrhage. An artery thus obstructed degenerates at last into a solid, small, impermeable cord.

The erysipelatous inflammation is not an idiopathic disease, but succeeds an injury, and extends along the internal coat until it arrives at the heart, when, of course, it must prove fatal. Mr. Guthrie has only been able to verify its existence after death in three cases.

"I have alluded to one case of this kind in my work on Gun-shot Wounds, page 96, third edition. The patient died suddenly and unexpectedly, the limb was greatly swelled and gorged with blood, the femoral artery, when opened, appeared more vascular than is commonly observed, its internal membrane was very red, and easily separated from the middle coat, and the fluid which lubricated its surface was of a more serous nature than usual, and greater in quantity. The other two cases I did not see until death was impending. In each, the right leg and thigh were greatly swelled and œdematous, the skin was of a pale dead white colour; the countenance was extremely anxious and bedewed with sweat; the pulse 140 and weak ; the patient sensible, and expressing hopes of recovery. In both cases the arteries showed the same character of disease, which extended upwards as high as the diaphragm. I gave a portion of the femoral artery taken from one of these cases of disease to Mr. Brookes, who considered it to be one of the finest specimens of arteritis he had ever seen. The symptoms which mark this state of disease, when distinguishable, and several cases are recorded where the appearances described have been noted, are a very quick pulse, a rapid deterioration of the state of the patient, and degeneration into irritative fever, with low delirium, followed by death.

In the three instances which I have mentioned, the erysipelatous inflammation of the arteries was evidently caused by continuity of parts with those already inflamed; and in all of them the inflammation was a deep-seated disease, situated in the muscles and internal structure of the thigh, the skin being apparently unaffected." 22.

Not having witnessed such cases we have nothing to remark, except that Mr. Guthrie is too well aware of the nature of the *staining* of vessels, to allow us to doubt of the accuracy of his conclusions. In the sloughing of the cellular tissue which seizes upon limbs after injuries in bad constitutions, nothing is more commonly observed than reddening of the inner tunics of the arteries, even to the heart. The symptoms are the same as those described by Mr. Guthrie, but the vessels are merely stained. So much for acute arteritis; and if men have been compelled by the difficulties of the investigation, or the strong counter-evidence of morbid anatomy, to acknowledge that they possess no positive information on its nature, nay, on its existence, still they have clung with almost foolish fondness to the idea, that we at least are sure of a chronic form of the disease. They have found, in the atheromatous and cartilaginous deposites of middle and advanced life, the data on which they triumphantly rely for confirmation of its prevalence.

Of these deposites, whether atheromatous or steatomatous, bony or cartilaginous, Mr. Guthrie gives a full and detailed account, which we particularly recommend to the attention of those who are anxious to become acquainted with morbid anatomy and the nature of disease. The subject is neither susceptible of analysis, nor sufficiently novel to require it. But, waiving mere description, we are met by the question of the origin and cause of these deviations from the healthy condition of a vessel. As we hinted before, our simplest notion of morbid causation is what we denominate inflammation—a more abstruse one, but scarcely less fashionable, is irritation. We neither quarrel with the names nor with the things, for we know full well how much more easy it is to destroy than to erect an edifice. But yet they can scarcely be considered as synonimous; for, although the one very commonly induces the other, and both do constantly occur together, yet, strictly speaking, the terms are intended to imply and convey an idea of dissimilar or distinct conditions. Mr. Guthrie, however, an able advocate of the inflammatory origin of the depositions in the coats of arteries, speaks of inflammation and irritation with an ambiguity and laxity of expression not at all characteristic of himself.

"Chronic irritation or inflammation, by which a slow change in the structure of a part is usually accomplished, appears to be the cause of the various alterations which are found in arteries. The first and simplest change is a loss of the elasticity natural to them, which may lead to a state of dilatation, without abrasion or rupture of any of the component parts of the artery, although sometimes accompanied by a general diminution of substance, and particularly of the middle coat. The cause which gives rise to the loss of elasticity seems, however, also capable of exciting in the artery a sort of preservative action by which it may be strengthened. The loss of elasticity is, therefore, more usually accompanied by changes which are very obvious; such as softening of the inner membranes, partial or general irregular thickenings, or depositions of cartilaginous, calcareous, or other matters, between the coats of the vessel. It is well known that irritation may give rise to alterations of structure of very different kinds; to the thickening of a part, to its partial removal by progressive absorption or thinning, and to its total removal by ulceration." 22.

Without going over the grounds which induce Mr. Guthrie to maintain the foregoing opinions, we may mention what induce us to dissent from the supposition of chronic inflammation being the essential cause of the deposites. As elasticity and suppleness of structure is the characteristic of infancy and youth, so is rigidity that of old age. The thigh-bone of a child, instead of breaking, will bend, or, like a green withy, one side will split, and the other will yield in such a manner as to be more or less doubled on itself. The bone of an octogenarian, on the contrary, snaps like a dry faggot. So it is with the cartilaginous structures, the intervertebral for instance; the adeps is absorbed; the cellular membrane is tough; the skin becomes a shrivelled hide; pellucid membranes, like the cornea, become opaque; the viscera are coated with cartilaginous-like capsules; the bronchi are bony; and, lastly, the arteries are rigid. Surely it is unphilosophical to select any one of these facts for a separate argument; and surely, also, if we look upon the whole, as we ought, it would be absurd to attribute the series of changes which the laws of the Creator have stamped upon his work, to the process of inflammation. Is an old man a mere conglomeration of chronic inflammations in every texture of his body? This would be physiology with a vengeance, and yet to such a dilemma are we reduced, if we attempt to single out this or that alteration as evidence of its existence. A modern and very able lecturer has asserted, that inflammation is growth, and growth is inflammation; if the present views be correct, the same must be said of decay; and thus from the cradle to the grave we are all, like so many whitlows, specimens only of acute and chronic inflammation. To be sure this sort of philosophy is simple.

Dropping any attempt at satire, which we really disclaim, we must say that the arguments advanced in support of the inflammatory origin of atheromatous and steatomatous depositions appear to be inconclusive and fallacious. The chief are, a redness and softening of the inner membrane of the artery. Neither are constant—the softening, in particular, is not even frequent. We have examined a considerable number of cases in which the arteries were diseased, and we are quite convinced that, however a low degree or kind of inflammation may be set up at times, *in consequence* of the irritation occasioned by the bony scales or atheromatous deposites, it has nothing to do with their formation. Setting aside the known indisposition of arteries to inflame, under circumstances highly favourable to the purpose, we think the manner in which the degenerations of the coats steal on, is strong presumptive evidence on our side of the question. Amongst hospital patients, it is rare to find the arteries perfectly healthy beyond the age of thirty-five or forty. The more muscular the subject, the more advanced are, cæteris paribus, the depositions; and, in confirmation of this view, we ascertain by the histories of cases, that men who have lived or worked hard are sooner affected than those who lead a quiet, or even a luxurious life.

In persons of laborious occupations, the system is worn out before its time, a sort of premature old age is established, the left side of the heart becomes hypertrophic, the arteries grow rigid early, those of the brain, in particular, become opaque or even bony; and dropsy, from the heart: aneurism, from the vessels: or apoplexy, from the cerebral arteries, crown that youth of labour, so lauded by poets, with a melancholy termination between the ages of forty and forty-five. This is a true picture, too faithfully drawn from the life, and not a composition, as painters say, from fancy. In the higher classes, the progress of the constitution's decline and fall is something different. The heart and the arterial system are still the sufferers, those arteries and that heart which have borne the brunt of fiery passions, the wear and tear of foolish fashionable dissipation. The mode of decay is, however, as we said, dissimilar from that which cuts off the hard-working mechanic. The arteries are not usually so soon nor so extensively affected; the heart, instead of becoming hypertrophic, grows large, flabby, perhaps loaded with fat; a disposition to yellowish fat appears about the trunk of the body; the vessels of the brain grow opaque and inelastic; and the face assumes a puffy and rather yellowish tint. These patients, on the whole, suffer less from dropsy than the lower classes, but are more affected with what is denominated angina pectoris; for this is almost, par excellence, the disease of the elderly nervous and luxurious invalid. To revert to the cause and origin of the depositions in the coats of the arteries, we maintain that there is no positive evidence whatever of chronic inflammation, or of any thing like it; whereas, an extended view of the changes which bodies undergo, from infancy to age, and dissolution, demonstrates conclusively, that their formation depends on those inscrutable laws which regulate the animal machine so long as the vital principle retains its fragile tenement. When we know what makes a tooth to grow and a tooth fall out; what covers the scalp of the babe with a flaxen down and the bald head of the sexagenarian with a few gray locks; then, and not before, can we explain the reason why the aorta of adolescence is as smooth as ivory, and that of old age as rough and chapped as the integument of icthyosis. It would be just as rational to imagine that chronic inflammation turns the hair white, as the arteries rough.

Before quitting the subject of the morbid alterations of the coats of arteries, we must advert to what has been denominated the "dissecting aneurism," a form of disease which Mr. Guthrie does not believe to be so rare as has been supposed.

"The simplest form is said to be that in which the internal and middle coats having given way, the external coat is only slightly separated by the blood effused beneath it. The chronic inflammation, which preceded this state, having however thickened the general surrounding wall of the artery, the separation of the external from the middle coat is effected with difficulty; the external coat becomes stretched and distended, and an aneurismal tumour is formed in the manner which has been so frequently described, although the accuracy of the description may be doubted. The separation to which I allude is much more distinctly marked, and the disease seems to have existed in the uniting medium, and between the coats of the artery, as much as in them: whilst the whole may be thickened, the separation is distinct.

In the first, or simplest kind, the tumour formed by the effused blood is circumscribed, and usually small in size. In the case to which I refer, it is otherwise; the blood is forced along the tube of the artery, separating in the simplest manner the external and middle coats from each other, and forming for several inches a long pouch or bag, which may or may not surround the vessel. In one case it formed a long pouch on the anterior part of the descending aorta, about six inches in length, extending to the sides, and in one place nearly surrounding it. A horizontal fissure, about half an inch in extent, near the upper part of the swelling, allowed the blood to pass through the inner and middle coats, and to effect this separation, which could only have arisen from disease previously existing in the part. This occurred many years ago, and I was not then aware that it was a peculiar or uncommon appearance." 41.

M. Laennec records a well marked instance of this affection, in which the external was more or less separated from the middle coat, from two inches below the commencement of the descending aorta down to the bifurcation of the iliacs. The sac was filled with blood and polypous concretions. Mr. Guthrie has met with and describes another well marked case of this description, the descending part of the arch of the aorta being affected. We now arrive at the section which treats of:—

### The Preternatural Dilatation of Arteries and Aneurism.

A clear yet comprehensive nomenclature of the different species of aneurism and dilatation is still a desideratum. It is astonishing what confusion has crept into the ideas of medical practitioners on this subject, for on making the examination of a body no two will name an aneurism alike. The partial introduction of Scarpa's views, has bred all this disorder, and the sooner they are discarded the better. The great stumbling block is the state of the internal tunics, some stiffly asserting they are ruptured in aneurism, others that they are not. Again, the varieties of dilatation are a bone of contention to systematic writers, and a bugbear to timid students; whilst the mode in which mere dilatation and aneurism, in the common acceptation of the term, run into one another, adds to the difficulties of the one and disputes of the other. We shall give Mr. Guthrie's ideas on this subject, that our readers may become acquainted with the results of his investigations in the Hunterian Museum, and other valuable fountains of information.

If the whole circumference of an artery be dilated, the coats of the vessel being more or less diseased, yet the inner tunic not so far destroyed as to have caused the deposition of lamellated coagulum, and obstructed the circulation, then this is called a *preternatural dilatation.* The arteries most frequently affected in this manner, are the aorta at its arch, and the innominata or carotids at their origin. It is not uncommon in the arteries within the cranium. We have said that in *preternatural dilatations* the coats of the vessel may be considerably diseased, nay, the inner coat may be fissured or abraded to some extent, and yet little or no coagulum is found attached to them. Sometimes, however, the tunics may be only thinner than natural, sometimes thicker and inelastic; we have seen them as dense and as tough as the sole of one's boot, and commonly in this state the internal surface is greatly puckered. When the preternatural dilatation has proceeded very far, coagula may be formed, but they are rarely lamellated, firm, or fleshy; not even when the inner coat is abraded.

When the tumour grows from one side or part only of an artery, it is called an *aneurismal tumour*, and this may proceed from a vessel in the state of preternatural dilatation. If no deposition of lamellated coagula has taken place, and if the coats be not more diseased than those of the artery, preternaturally dilated or not, from which it has arisen, we should prefer terming it an *aneurismal dilatation.* If, however, the coats be more destroyed, and lamellated or firm coagula exist in it, we should call it at once an *aneurism.* We are aware that the two latter differ more in fortuitous circumstances, than in nature or even degree, but still we think the definitions we have proposed would be more precise, than when both states are indifferently styled, aneurismal tumour.

It is not necessary, as Mr. Guthrie well observes, that the inner tunic must be removed or even materially affected in an aneurism, before the deposition of coagula takes place. This error, which derives its origin from Scarpa, must be exploded before we shall make any progress in our pathological notions of the disease. It appears, says Mr. G., that this change, the deposition of coagula, occurs *with the commencement of the alteration of the function* of the inner coat, and certainly before its removal by absorption. This explanation goes for nothing, for one aneurismal dilatation will shew the lamellated coagula, whilst another in a similar condition, as far as the tunics are concerned, will have none. The truth is, that our knowledge on this subject is extremely imperfect, although it may safely be received as a general rule, that the more the inner tunics are destroyed, the greater will be the likelihood of the deposition of lamellated coagula.

In large aneurisms the coagulum suffers a material change in the course of time, undergoing a species of decomposition, which perhaps leads at last to the insinuation of blood between the layers and to the ultimate rupture of the sac. Sometimes in old aneurisms it is quite opaque and firm, at other times partly transparent, and resembling horn or glue a little warmed and softened before the fire. In some cases, further changes will be found to have taken place exterior to this, and the matter deposited is softer, more easily broken up, and offering little opposition to the passage of blood through it. In some very rare cases of preternatural enlargement of the aorta, and of other vessels, the coagulum is found around the sides of the artery, leaving a passage for the blood through its centre as a proper canal. The preparation, No. 372, of the Hunterian Museum shows this very rare occurrence.

"When the walls of an artery yield by dilatation in any spot, not including the circumference, but (as is usually the case) on one side of the vessel only, and frequently in a small space, it is called a *true aneurism ;* the internal and middle coats being found perfect on examination, even by maceration. This state of disease is for the most part only seen distinctly in small aneurisms, for as they increase in size, the inner and middle coats appear to be removed by absorption ; they may perhaps in some cases be ruptured. The first step in the formation of an aneurism in a part of the aorta which is even preternaturally dilated, is the deprivation of a greater portion, if not of the whole of the remaining elasticity which the part possessed. This is followed, if not accompanied, in general by the deposition of a curdy yellowish matter at the spot, which now becomes dilated to the whole extent of the deposit, and this probably regulates the size of the opening between the aneurismal sac and the artery generally. The dilatation is usually at first more or less circular or oval, from the size of a large pin's head upwards, to any extent the artery will admit of. In some instances it seems to form a sort of split or fissure rather than an oval opening. If an aneurism of the size of a pea, or of the end of the finger be examined, by making pressure around it ; a small quantity of the yellowish curdy matter may frequently be pressed out from under the inner coat, which yields to allow it a passage. If a careful dissection be made from without inwards, the three coats may be always distinctly shown, and this same yellowish matter demonstrated as dependent on the middle coat. From the first moment that the aneurismal dilatation takes place, and before it is large enough to admit the end of the little finger; it becomes filled with a soft coagulum, forming a striking difference when compared with the enlarged but empty preternaturally dilated aorta in which this little aneurism is situated. From the moment the spot yields, so as to form the commencement of an aneurismal sac, the edge of the artery surrounding or enclosing this sac becomes thicker and firmer, so as to form a distinct thickened, yet well defined although rounded edge. This edge seems to be the product of a healthier inflammation than that which has given rise to the deposit of the atheromatous or yellowish curdy matter alluded to. It is set up by nature to form a boundary to the mischief, as healthy inflammation is established in other parts of the body, previously to the formation of a line of separation between those which are mortified and those which are sound. Over this edge the inner and middle coats of the artery can always be traced; and even throughout when the sac is small, and for some distance beyond the edge when it is large. In this last case they soon become confused, and are often so blended together as not to be traced; although at others the termination of the inner coat may be seen, as if it had been irregularly removed or torn. It is only then in small aneurisms that the structure of the sac can be fairly traced; for when they have attained a large size, the aggregation of matters external to them, and the slow but continued action which is going on, render a distinct separation of the component parts of the sac impossible.

When the aneurism has increased in size to that point at which the inner and middle coats begin to be removed from the centre of the tumour, the external coat forms the sac. It does not usually remain of its natural thickness, or is simply dilated and thinned. On the contrary, from the commencement of the separation, and indeed before the removal of the internal coats, some degree of increased or altered action has taken place, which appears to be afterwards augmented. The pressure from the enlargement of the sac gives rise to irritation and the formation of adhesions with the surrounding parts, as if nature endeavoured to prevent the mischief impending from rupture. As soon as the sac meets with an opposition to its dilatation, arising from harder or more resisting parts, absorption begins; the sac becomes thin and is ultimately removed. The hard parts, such as bones, follow by that process of absorption called by Mr. Hunter progressive, in which that action of the small arteries necessary to constitute inflammation is wanting; there is therefore little comparative pain at the commencement of the process, and no formation of matter. Cartilages from their elasticity resist this absorbing process more successfully than bones; those of the ribs will remain after the bone has been removed; and the intervertebral substance appears to be unaffected, when the bodies of the vertebræ have nearly disappeared. After the hard parts have been removed, the progress of the aneurism is more rapid. The skin becomes distended, loses its furrows, and assumes a shining white appearance. This is soon changed to a copper-coloured hue: some one or more parts of the external tumour become elevated: the skin over them becomes dark coloured, and an evident slough is formed. This slough does not yet yield altogether; blood begins to ooze at the edge in one or more points, and slowly increases in quantity until it gradually destroys the patient; or a sudden and more rapid discharge at once closes the scene: a catastrophe which may in some instances be uselessly delayed, by supporting by compress and bandage the part which seems to be the weakest and the most likely to yield. When an aneurism protrudes into a cavity lined by mucous membrane, the process is much the same; but it is said that the opening which takes place into any cavity or canal lined by serous membrane, occurs by rupture and not by sloughing. In some very rare cases, when an aneurism approaches to and gives rise to inflammation of the periosteum covering the bones, ossific matter is thrown out, forming a sort of wall to the tumour, and impeding its progress in that direction; but I suspect a peculiar diathesis to exist under such circumstances." 58.

The foregoing description is extremely accurate, but we would abolish the designations of "*true*" and "*false* aneurism" as here applied, and substitute "aneurismal dilatation," and "aneurism" for them. The meaning we have attached to the latter terms we have already mentioned. If an aneurism be formed upon a preternaturally dilated artery, it sometimes happens that the walls are thin, become dilated in consequence, and give rise to pouches or sacculi proceeding from the aneurism itself. Although aneurism for the most part argues a generally diseased state of the arterial system, yet it not unfrequently may exist in an extremity, and an operation be attended with a permanent cure.* Mr. Guthrie knew a man who survived the operation for popliteal aneurism in both limbs for twenty-five years, and died of fever at last. It has been stated lately that no vessel is given off from an aneurismal sac, but in many of the old operations vessels opened *into* it, and when there is a preternatural enlargement, the circulation will go on through it, and the arteries will arise from it.

Mr. Guthrie enters into a very just though spirited critique of the opinions and statements of Scarpa, opinions and statements which have done much good and much harm. For the criticisms in question we must refer to the work itself, and shall content ourselves with the following short quotation.

"There are many very fine preparations in the Hunterian collection, which appear at first sight to support the opinion maintained by Scarpa, that a rupture takes place of the inner and middle coats of the artery in cases of aneurism; but a careful examination of them rather leads to a different conclusion. The oval, rounded, and even fissure-like opening would naturally seem to be the result of a rupture, such as has been described under the head of a dissecting aneurism, at page 43; and it is by no means intended to deny that this accident may occur in some instances. I have seen the inner coat, and even the middle one ruptured, in cases in which these parts had been previously softened by inflammation. The appearances observed on the dissection of the body of His Majesty George the Second are a proof of the occurrence, if others were wanting. Nicholls says, a fissure was found on the inner side of the trunk of the aorta, about an inch and a half long, from which some blood had recently passed under its external coat, and formed an elevated ecchymosis. These fissures or rents are usually found to take place in the circular direction of the artery, and in the course of the semicircular fibres of the middle coats, which may possibly account for their looking as if cut by a sharp instrument. They have been, although rarely, met with running in different directions; and when the parts have been ruptured irregularly, the rugged edges have been supposed to adhere to the outer coat, or to be gradually rounded off by time, so as to form the smooth, round, or oval opening, which is so frequently seen to be the means of communication between an aneurismal sac and the canal of the artery. Yet the gradual formation of this oval or rounded opening may be so easily traced from the atheromatous patch to a complete hole or fissure, that the evidence appears much more complete in favour of the dilatation followed by absorption of the inner and middle coats in internal aneurisms, than it is in favour of a rupture of them in the first instance. The last part of the argument, *viz.* whether these coats, and especially the inner one, do or do not pass over the rounded edges of the opening, is an appeal to a fact which appears to be more easily demonstrable

---

* It is in discriminating the cases in which the heart and great vessels continue sound or otherwise, that the stethoscope proves of the most essential service.

than it really is; still the preparations alluded to seem to demonstrate the fact in most instances in which they have been examined, or only to leave it doubtful from the point not having been sufficiently attended to. The truth is, that the subject has not yet been satisfactorily investigated; and the authority of Scarpa has perhaps hitherto prevented due inquiries being made, so as to leave, from a multiplicity of observations, no doubt of the real nature of the case. The preparations 388, and 367, A, deserve attention, being instances of the doubtful manner in which the aneurism has been formed." 77.

When an aneurism is formed through the rupture of the middle and internal coats and distention of the external, it is called a *false* aneurism. Where a *true* aneurism, i. e. a dilatation, after attaining a definite size, suddenly increases at one spot, so as to give the tumour the appearance of one swelling above another, it is usually called a *mixed false* aneurism; and in this the opening into the second pouch is either by absorption of the inner coats or by rupture. The inner coat has been said to protrude between the other two, and to form a sac of an aneurism, called *mixed internal.* Of this a dubious specimen has been reported to have been observed in Paris; but experiments, so far as they go, are against it. When an artery, however, has been wounded, the cicatrix is apt to dilate and constitute a kind of true aneurism. Of this our author believes that he once saw an instance, in an officer whose carotid artery at its bifurcation was injured by an arrow in India.

When aneurism is the result of an accidental affection, and the tumour has increased to some magnitude, the external coat is ruptured at an early period, and does not usually form the sac. This is produced by the blood effused, and the consolidation of the neighbouring parts. It is called a *spurious external circumscribed* or *diffused* aneurism, as the case may be. On examination, the ruptured part of the artery is readily distinguished, and a portion of the artery is usually deficient, for nearly, if not the whole of its circumference. The vessel is commonly affected with the atheromatous or steatomatous patches above and below the immediate seat of the aneurism. When an artery is wounded, and the external opening in the integuments closes so as to prevent the blood flowing through it, a *traumatic spurious external circumscribed* or *diffused* aneurism results, according to the facility which the neighbouring parts offer for the confinement or diffusion of the extravasated blood. Thus, traumatic aneurism differs materially from the spurious, in the circumstance of the coats being sound in the former and unsound in the latter. Mr. Shekelton has described in the Dublin Hospital Reports another species, closely resembling what has been denominated the *dissecting* aneurism. The blood forced its way through the middle and inner coats, and dissected the middle from the outer for the space of four inches, when it again forced its way into the canal of the artery, through the middle and inner coats.

We have given the foregoing nomenclature without note or comment so far, but here we must pause and protest that it forms a crabbed, and almost unintelligible jargon, instead of a simple and scientific system of names. A traumatic spurious external diffused aneurism! Why, good heavens! it reminds one only of those concise pieces of description to be found in the classics of the nursery! Mr. Guthrie, of course, is not to blame, for he has not ventured to coin these villainous hard words, but merely offered them as current terms;—indeed, we suspect that he feels no greater relish for their use than we do. To the mode in which *true* and *false* aneurism are employed we have already objected, and therefore need say no more on that point. The *spurious* aneurism we would abandon altogether, as it does not differ one jot from ordinary aneurism. *Circumscribed* and *diffused* are useful distinctions; *external* is unnecessary, cumbrous, and bad, as the particular affection is always expressed by the name of the vessel affected. *Traumatic* aneurism is a proper expression, an indispensable distinction; and circumscribed or diffused are also indispensable epithets to be added to it. To conclude then, we advocate simplification as far as possible, and we must repeat that the present nomenclature, correctly and properly stated by Mr. Guthrie, is vicious, laboured, and clumsy.

After a short notice of the *causes* of aneurism, Mr. G. enumerates the different modes in which a *spontaneous cure* of the disease may take place. They are supposed, as our readers well know, to be three; *viz.*—1. By coagulation of the contents of the sac. 2. By sloughing. 3. By an accidental pressure of the sac upon the artery. With regard to the first, our author observes that nature adopts two means to favour it: the enlargement of the collateral branches, and an attempt to close or shut up the lower openings into the aneurism or those most distant from the heart. On the latter point Mr. Guthrie mentions some interesting facts. In a case of inguinal aneurism where Mr. White operated below the tumour, the canal of the artery in that situation was found contracted to one-fourth its natural size. The sac sloughed, and the man died.

In the Hunterian collection are several instances of all the openings into an aneurism, except the upper one, having been closed during life. These preparations show also that when the lower end of the artery has been obliterated the *aneurism has not ceased to increase.* Such a step, when once accomplished, or even begun, undoubtedly favours the coagulation of the blood, at least under certain circumstances; but to institute a comparison between it and ligature of the vessel above the tumour, is chimerical and absurd in the highest degree. The preparations, No. 386, 392, and 397, are sufficient evidence on this subject, not to mention the many cases to be found in authors.

"Nothing is more simple than to say that the blood coagulates in layers, or as a whole, until the sac is filled with it; when nature considers it as an extraneous substance, and employs the absorbents for its removal until the artery is rendered an impervious ligamentous cord, having attached to it a small hard fleshy tumour, which was the aneurism. It is more difficult to explain why this does not always take place, than why it should occur in particular instances. In these cases of spontaneous cure, accompanied by obliteration of the artery, in which dissection has enabled us to ascertain the state of parts, it has always been found that the coagulum of blood was continued into the upper portion of the artery. It is possible, that nature having in some instances closed the lower opening of an aneurism, endeavours to do the same with that nearest the heart. It is perhaps this effort which gives rise to the formation of the coagulum which fills the tumour; an effort which must always be opposed by the impetus of the blood, unless the coats of the artery take on that higher degree of inflammatory action which leads to effusion on the inner membrane, and the establishment of a power capable of resisting the force of the blood, when the spontaneous cure would be effected. It is very likely then, that the spontaneous coagulation of the blood in an aneurismal tumour is accompanied by a change in the action going on, either in the artery or in the walls of the sac, or in both." 94.

Mr. Guthrie doubts the reported influence of one aneurism in assisting the cure of another below it, and refers to cases and preparations in disproof of it. In illustration of the cure of aneurism by *sloughing* of the sac, Mr. G. relates the case that occurred to Mr. Albert in the York Hospital. The tumour, which was of large size and extended below and above Poupart's ligament, sloughed entirely out, and gave issue to several pounds of coagulum, after which granulations sprang up, and the sore ultimately healed. In two other cases the patients died, worn out by the discharge and extension of the ulceration to the hip-joint. The operation, says our able author emphatically, should *never* be had recourse to after mortification has begun. Mr. Guthrie is not satisfied of the accuracy of the opinion regarding the cure of aneurism, by the pressure of the sac on the artery above. Neighbouring vessels have been obliterated in this way, but Mr. G. has never seen such a method of cure, nor, as far as he knows, has it ever been proved by dissection. Mr. Guthrie relates a case with the view of shewing that dilatations of arteries may subside. He is not exactly convinced by the details, nor are we, and we therefore pass it without further mention.

On the symptoms and diagnosis of aneurism Mr. Guthrie has little novelty to offer, nor is it rational to expect any. To one point, however, we may advantageously direct the attention of our readers. An abscess may form between the skin and the sac, and give rise, if not early opened, to external inflammation and ulceration. The danger of these cases, which are fortunately rare, consists in the sac being usually opened by the ulcerative process. Breschet, in the notes to his translation of Hodgson, relates a remarkable case. A child, æt. 10, was affected with several abscesses after an attack of fever. Then a tumour appeared on the anterior and superior part of the thorax, which had all the characters of abscess, and was punctured as such. A jet of blood followed and continued till the tumour was emptied, when an irritating injection was thrown into the sac. The hæmorrhage was not arrested, the tumour began to pulsate, and in two or three days the little patient died. On dissection, the tumour was found to consist of two parts, one exterior to the sternum and the other within it, the two communicating through an opening in the bone, which was altered greatly in structure and crumbled away on being touched. At the bottom of the internal cavity, the anterior surface of the aorta was seen, the tumour lying upon it. It was not in the least dilated, but on the anterior part of the arch near the innominata was a small perforation one line and a half long, and one wide. We should think, with Mr. Guthrie, that the opening into the artery was a secondary process.

Warner relates a curious case of pulsating tumour between the two portions of a fractured sternum. It had most of the usual characters of aneurism, but being left to take its course, it burst in three weeks, and discharged a considerable quantity of matter, after which the patient did well. Surgeons should recollect that arteries may occasionally take unusual courses, and so communicate pulsation to abscesses or tumours remote from their common situation. Lumbar or psoas abscess may, under certain circumstances, simulate aneurisms. If, however, the patient be placed in the recumbent posture the tumour is diminished or removed, when the course of the artery is more readily and accurately examined.

The *medical treatment* of aneurism comes next upon the tapis, but as Mr. Guthrie's sentiments on this occasion do not differ from those of all modern well-informed practitioners, we need not stop to examine them in detail.

The section on the COLLATERAL CIRCULATION deserves and must obtain more particular notice. Mr. Guthrie advances propositions, which, however startling they may appear to some, we believe to be worthy of the most deliberate and careful investigation. Until within the last thirty years the powers of the collateral circulation were undervalued, indeed unknown. The scepticism and ignorance of the older surgeons were fostered and nourished, if not originally created, by their own injurious and clumsy operations. Their modes of including in one knot vein, artery, nerves, or whatever was in their way, were the surest means of producing the mortification which they dreaded. Mr. Guthrie, however, judiciously observes, that more modern surgeons may perhaps have run into an opposite extreme, and exchanged the doubts and fears of their forefathers for an overweening and dangerous confidence. In plain terms, they have looked at the collateral circulation through the magnifying glass of modern innovation, and run riot in their presumption on the powers of nature. In all cases, says our eloquent author, in which the circulation is not fully re-established at once, there is a period of doubt, in which a sort of contest is carried on by Nature for the life of the limb, in which she is sometimes successful, often the reverse.

The collateral circulation is more perfect and powerful during the increase and growth of the body, than either at maturity or the decline of life. This observation has been founded on experience, and is readily explicable by theory; but it must also be remembered, that the nature of the disease or injury, and the period that has elapsed between the operation and the infliction of one or commencement of the other, are circumstances that exert a commanding influence. On dissection of persons on whom the operation for aneurism has been successfully performed, we find that arteries known to exist previously have become very much enlarged, and that others not perceptible in the sound limb have been developed in a similar manner. The question is *when* do these vessels begin to assume their unusual size? In other words, *when* does the establishment of the collateral circulation commence?

There are evidently two kinds of communication between arteries:—the first by direct branches of a certain size, as between the palmar and plantar arteries; the second by the inosculations of the capillary vessels. When a vessel under circumstances of the former description is wounded, red blood flows from each cut end; when under the latter, the blood from the lower orifice is dark-coloured and venous. This proves, in Mr. G.'s opinion, that the collateral circulation is carried on, in the first instance, by blood drawn in part from the veins as well as the arteries.

"If a limb be injected and dissected most carefully, four or five days after a ligature has been placed high up on the principal trunk, the capillary vessels will be seen to be well injected; but few or none will be found large enough to admit of the inosculation being traced throughout. If another limb be injected and dissected, forty days after the ligature had been applied, (and these experiments have been made on animals, and opportunities have occurred of making similar dissections on man) a difference will be distinctly observed between the two preparations. In the latter, the capillaries will not appear to be so fully injected, but several larger and more tortuous vessels will be found in situations where they were not expected to exist; and the anastomoses of these one with another, and generally by arches, may be traced to their communication with the principal trunk, both above and below the obliterated parts. Let an incision now be made in the nearest pervious portion of the lower part of the artery, and red arterial blood will issue from it. The communication has become direct by communicating branches, and the capillaries have returned to their accustomed duties. After the lapse of years, these communicating branches will again appear to have diminished their numbers, one or more will have become predominant, forming large direct canals of communication, and the smaller will have shrunk up, or be no longer discernible. The limb has in fact returned to its natural state." 136.

During the first twenty-four hours after the ligature of a large vessel, the temperature of the limb is always diminished; after that period it is commonly from three to five degrees higher than in the other; and at the end of from eighteen to twenty-eight days, the temperature is alike in both limbs. These facts, and the great enlargement which small vessels undergo, prove that the collateral circulation is necessary, is absolutely indispensable, to the maintenance of the life of the extremity. We revert then again to our former interrogatory, *when* does it begin to be established?

"Does it commence with the disease, take place during its progress, or only after the operation? I am of opinion, that the collateral branches begin to enlarge shortly after the commencement of the disease, as a part of the curative process which nature endeavours to set up in most instances; the essential points of which are, in an extremity, 1st, the obliteration of the artery above and below the tumour; 2d, the coagulation of the blood within it; 3d, the enlargement of the collateral branches above and below it.

When a limb is lost through mortification, as the consequence of a division or obstruction of the principal artery, it usually takes place after the infliction of a sudden injury, in consequence of the collateral branches not having had time to enlarge.

If the femoral artery be punctured near the groin, and a diffused aneurism form in a few days, extending up to Poupart's ligament; can the operation of placing a ligature on the external iliac be performed on the same principle, or with the same hope of success, as if the case had been one of true aneurism of several weeks or months formation? The answer is in the negative. The present theory of aneurism is not applicable to the case. The surgeon who placed a ligature on the external iliac, would probably lose his patient from mortification, because the collateral branches would not yet have had time to enlarge.

When an aneurismal limb has been injected, on which an operation has not been performed; the collateral vessels have all been found larger and more fully shown than on the opposite side, although not to the same extent as in cases of a similar nature in which the operation had been done.

It is necessary that this enlargement of the collateral branches should take place, because in many cases the artery beyond or below the tumour is obliterated long before any operation is performed. The main supply of blood is already cut off from the extremity, and the operation adds very little to the derangement of the circulation which has already taken place below the tumour.

These facts appear to me to be conclusive: they show that the collateral circulation is not the same, is not in the same stage of preparation, in a limb suffering from a divided artery, as in one in which an aneurism has for some time existed; and they also show why mortification is more common after wounded arteries than after operations for aneurism." 140.

The practical conclusions deduced by Mr. Guthrie, from these premises are:—that the theory of the operation for ordinary aneurism cannot be applied with safety to aneurisms dependent on wounded arteries;* that it is inapplicable to wounded arteries; and that the length of time an aneurism has existed is, cæteris paribus, a guarantee of safety as far as the collateral circulation is concerned. Mr. Guthrie remarks that in the upper extremity the collateral vessels are, as a general rule, capable of carrying on the circulation under *any* circumstances: but that when the femoral artery is suddenly cut across, mortification is not an uncommon consequence, especially in elderly persons. If the femoral vein be also divided, Mr. Guthrie believes that mortification seldom fails to follow. We have had opportunities of being convinced of the general accuracy of these deductions, and we therefore wish to impress them most earnestly on the minds of students, and of our more experienced brethren.

The section on the Surgical Treatment of Aneurism is interesting in a historical point of view, and no less so because our author boldly and generously vindicates to Mr. Hunter, the claim of having originated by the force of his genius the modern operation for its cure. Mr. Guthrie has shewn that the successors of Anel and the contemporaries of Dessault, made no attempt to deck their brows with the laurels which were worn by John Hunter. It was reserved for the jealousy of more modern French surgeons to attribute the Hunterian operation to the celebrated men alluded to; a jealousy which has defeated its own aim, and, to use a coarse expression, more firmly fixed the saddle on the proper steed. We part from this unpleasant subject without regret. It has occasionally happened that the operation for popliteal aneurism has proved unsuccessful, either from the sac being supplied with a duplex femoral artery, or from large collateral vessels having carried sufficient blood into the artery above the sac to prevent the coagulation and absorption of its contents. Such a case happened at St. George's Hospital; amputation was performed, and the patient died. In another instance which lately occurred to Mr. Briggs, a different mode of practice was adopted, and with complete success. The case is short, and requires no abridgment.

"James Mack, carpenter, aged thirty years, in February, 1829, felt pain in the toes and ankle and knee, and afterwards a swelling like a pigeon's egg in the ham, which gave him great pain, particularly at night, and pulsated like his heart. The operation was performed on the 6th of March, by Mr. Briggs. The ligature came away on the 27th, the pulsation in the tumour ceased, and it diminished very much in size. It was only in June he was able to go to his work, when the swelling had entirely disappeared. In September it began to appear again with pulsation, both the swelling and pulsation being soon greater than before. A compress was applied with a bandage on the part and on the leg, which made it worse. He then applied a hard compress, being in fact a narrow roller four inches in length, on the inside of the thigh, just above the knee, and above the inner hamstring muscles, which was firmly retained in that

---

* This passage is applicable only to the *diffused* aneurism from a recent wound.—*Rev.*

situation night and day. This gave him relief by taking away the pain, which was principally felt in the toes and ankle. This compress he wore for two months, at the end of which time the pulsation had ceased, although the swelling had not entirely subsided. He has gradually improved ever since, although he has still pain in the back of the leg, and on walking he feels the foot, and particularly the great toe, numb and cold. He cannot quite straighten the limb, and walks with a little halt. There is no appearance of the swelling now, one year after the operation." 157.

The description of the effects of a ligature on an artery need not detain us, as we stated Mr. Guthrie's experiments and observations on this head, in our article on wounds and injuries of vessels. To that article we refer our readers. The division of our author's work expressly dedicated to the subject of aneurism closes with a full, and we will add, very masterly consideration of the operation of tying the vessel beyond the tumour. Our own opinions on this subject are before the public, and hitherto we have seen no reason to modify or retract one single iota of them. We should feel great pleasure in putting our readers in possession of Mr. Guthrie's sentiments and reasonings on this subject, if we were not convinced that it has been argued latterly ad nauseam. As journalists, we must consider not only what is good in itself, but what is palatable, for no where is *toujours perdrix* more sickening than in periodical literature. When a question has been worn nearly threadbare, the best way is to put it by for a season, to let it lie fallow for a year or two, and then it comes out with all the gloss of novelty, and all the productiveness of repose. Anel's operation was forgotten until Hunter's revolutionized surgery. Brasdor and Deschamps had tied arteries beyond the tumour without success, and their operation was quietly slumbering in cobwebs and oblivion, till Sir Astley Cooper revived it, and Mr. Wardrop became its Coryphæus. At some future day our posterity may perchance be astonished to find that in the work of an old and valuable author of the name of Guthrie, the merits of a proceeding are elaborately discussed, that had just been *invented* by a Wardrop yet unborn.

Here we must conclude, and were we used to the language of panegyric, we might add a laboured eulogy on Mr. Guthrie's Work. But we will not attempt what would neither enhance his merits nor our honesty. We have read the work with diligence, and have derived from it information of such a quality, that we hope, please God, to read it yet again. It is not to be supposed that in a volume of 416 pages on such a subject, every page is to teem with novelty, every chapter with a discovery; he must be a very thoughtless or unreasonable man who could expect it. But the principles inculcated are sound, the observations judicious and frequently indicative of deep reflection, the research great, the experience ample, the deductions practical and acute. If those be not strong recommendations to a work, we know of none; if this will not suit the profession, we would advise it to purchase the lucubrations of Doctor Haslam, and the practical remarks of Mister Wakley.

